# Gut microbiome profiles in Thai healthy pregnant women and its association with types of foods

**DOI:** 10.1186/s12884-022-04397-5

**Published:** 2022-01-29

**Authors:** P. Phoonlapdacha, C. Tangshewinsirikul, J. Phosuwattanakul, K. Kittisakmontri, S. Nitisinprasert, J. Nakayama, P. Prombutara, U. Suthutvoravut, N. Chongviriyaphan

**Affiliations:** 1 Division of Nutrition, Department of Pediatrics, Faculty of Medicine Ramathibodi Hospital, Mahidol University, Bangkok, Thailand; 2 Division of Maternal Fetal Medicine, Department of Obstetrics and Gynecology, Faculty of Medicine Ramathibodi Hospital, Mahidol University, Bangkok, Thailand; 3 Division of Nutrition, Department of Pediatrics, Maharat Nakhon Ratchasima Hospital, Nakhon Ratchasima, Thailand; 4 Department of Pediatrics, Faculty of Medicine, Chiang Mai University, Chiang Mai, Thailand; 5 Department of Biotechnology, Faculty of Agro-Industry, Kasetsart University, Bangkok, Thailand; 6 Department of Bioscience and Biotechnology, Faculty of Agriculture, Kyushu University, Fukuoka, Japan; 7 Omics Science and Bioinformatics Center, Faculty of Science, Chulalongkorn University, Bangkok, Thailand

**Keywords:** Gut microbiome, Pregnancy, Dietary intake, Glutinous rice

## Abstract

**Background:**

Gut microbiome colonization during early life is significant for immunological and physiological development. Maternal microbiome is associated with proper development of infants. The aim of this study was to determine the gut microbiome profiles among Thai healthy pregnant women and its associated factors.

**Methods:**

A multicenter, open trial prospective study was performed at three hospitals in Northern, Central, and Northeastern regions of Thailand. Thai healthy pregnant women attending antenatal clinics were recruited. Fecal samples of subjects at the third trimester of pregnancy were collected with sterilized techniques. The gut microbiome profiles and bacterial diversity were assessed using 16Ss RNA gene sequencing. Demographic data, dietary intake, and anthropometric data were recorded and analyzed.

**Results:**

There were 86 healthy pregnant women. The dominant of gut microbiome profiles were Bacteroidetes and Firmicutes. Pregnant women in the Central region had significantly higher of Ruminococcaceae and Lachnospiraceae than those in other regions (*p* < 0.001). Pregnant women in the Northern region significantly consumed more glutinous rice than those in other regions (*p* < 0.001). Glutinous rice intake was positively correlated with Bacteroidetes (rho = 0.405, *p* = 0.01) and negatively correlated with Firmicutes (rho = − 0.440, *p* = 0.001). Alpha diversity was not correlated with pre-pregnancy body mass index (BMI) or gestational weight gain.

**Conclusions:**

The gut microbiome profiles mainly found in Thai healthy pregnant women were Bacteroidetes and Firmicutes. The gut microbiome profiles in pregnant women found in this study possibly depended on dietary patterns. Glutinous rice with high amylopectin is probably related to abundance of Bacteroidetes.

**Supplementary Information:**

The online version contains supplementary material available at 10.1186/s12884-022-04397-5.

## Background

Pregnancy is a stage of several changes including hormonal, metabolic, and immunological parameters that affect maternal health and facilitate optimal fetal growth and development [1]. There is evidence showing that the maternal microbiome impacts the development and health outcomes of offsprings during prenatal and early postnatal periods [2, 3], but the mechanisms are unclear. There are a few studies of the profiles of gut microbiome in pregnant women [4]. In addition, there is the discrepancy in the nature of microbiome changes during pregnancy [3, 5, 6]. The differences may be explained by the difference in various factors such as gestational age, genetics, ethnicity as well as environmental factors. It has been shown that maternal diets [7–9], pre-pregnancy weight, and weight gain during pregnancy [10–12] contribute to the composition of the microbiome during pregnancy.

The composition and diversity of gut microbiome is affected by many factors including diets [13]. Diet consumption varies due to the differences in agricultural and cultural practices in different countries. The major issues of practice variation include types of food, meal preparation, drinks, desserts, cooking utensils, and performance or religious ceremony [14]. Eating habits may be varied among people living in the same geographical areas or living in different areas, such as urban and rural areas, within the same country or having different socio-cultural practices.

It has been shown that variation in feeding behavior has a strong influence on the composition of gut microbiota [15]. *Bacteroides* enterotype increases in the intestinal tract of people living in Western countries, eating western diets high in fat and protein. On the other hand, *Prevotella* enterotype is common in non-Western countries where their people consume diets high in fiber [16]. De Filipo et al. reported an abundance of Bacteroidetes, Actinobacteria, and Enterobacteriaceae in rural African children, whereas Firmicutes were predominantly present in European counterparts. It has been shown that the geographical difference in microbiota diversity may be due to the variation between the modern Western diets consumed in Italy and the African diets consumed in the rural Burkina Faso [17]. Wie et al. reported a powerful link between ethnicity as well as socio-economic status and bacterial diversity in the Southeast Asia [18].

According to the geographical areas, Thailand is a country in Southeast Asia. One unique feature of Thai culture is Thai food. Thai food is full of diversity. It has a lot of menus each distinctly classified into 4 regions: namely Northern, Northeastern, Central, and Southern. The food in each region has its own identity according to the culture, and availability of raw materials indigenous to the area. A Thai meal is predominantly comprised of rice. Side dishes eaten with rice commonly consist of vegetables, meat, seafood, fish, and eggs. Thai foods are different from the foods consumed in other regions of the world, resulting in the difference in the gut microbiome between Thai people and those from other countries.

In Thailand, La-ongkham et al. showed that children in the Northeastern region had a high consumption of meat (chicken and beef) and a variety of carbohydrates (noodle, fermented rice, and sweet potato) as well as vegetables and fruits. On the other hand, rice, breakfast cereal, and cow milk were significantly preferable in children in the Central region. The authors showed that the gut microbiota profile of children in the Northeastern region was significantly abundant in lactobacilli, *Clostridium coccoides*–*Eubacterium rectale*, *Clostridium leptum*, *Prevotella,* and *Bacteroides fragilis* [19].

Gut microbiome is related to maternal health during pregnancy and fetal outcomes, and variations of diet influence its profiles. To our knowledge, there is no report on gut microbiome profiles in Thai pregnant women. This study was conducted to determine the gut microbiome profiles among pregnant women with different eating habits and food types in three regions of Thailand.

## Methods

### Study population

This study was a part of a multicenter prospective cohort study of the gut microbiome profiles in infants aged 0-1 year. Healthy pregnant women aged 18-42 years from three different geographic regions of Thailand as follows: Northern (Suandok Hospital, Chiang Mai), Central (Ramathibodi Hospital, Bangkok), and Northeastern (Maharat Nakhon Ratchasima Hospital, Nakhon Ratchasima) were enrolled. The study was performed during December 2015 to June 2017. Exclusion criteria were having alcohol consumption more than 7 units per week (one unit equals to a glass of beer, wine, or a measure of spirits), receiving antibiotics within 2 weeks of fecal sampling, and receiving immunomodulatory drugs within 4 weeks prior to delivery. The study protocol was approved by Committee of Human Rights Related to Research Involving Human Subjects, Faculty of Medicine Ramathibodi Hospital, Mahidol University (ID10-58-16). All participants provided written informed consent before enrollment.

### Data collection

Demographic data including maternal age, religion, socioeconomic status, and maternal education level were collected using questionnaires. Antenatal and perinatal data were reviewed from medical records of mothers who had antenatal care and delivered their babies at the study hospitals. For mothers attended antenatal care and delivered elsewhere, Thai handbooks of mother-and-child health were reviewed. Anthropometrics were assessed by using standard digital scales. Pre-pregnancy weight, gestational weight gain, and gestational age were collected using hospital records. Pre-pregnancy BMI was calculated by dividing weight (kg) with height square (m^2^).

### Dietary data

Dietary intakes were collected by a nutritionist using semi-quantitative food frequency questionnaires (FFQ) (Supplementary Table 1), which was constructed to focus on food sources of fiber, prebiotics, and probiotics. Moreover, as rice is the main carbohydrate source for Thai people, we categorized by type of rice as white rice and glutinous rice. Participants provided the frequency and the average quantity of each food item consumed during 4 months before data collection. Three non-consecutive (2 weekdays and 1 weekend) 24-h dietary recalls were obtained by a nutritionist to determine energy and macronutrients intakes of all participants using 24-h food record forms. Dietary intakes of energy, macronutrients, and fiber intakes were calculated using the INMUCAL-nutrients software of Institute of Nutrition, Mahidol University [20], and compared with Thai Dietary Reference Intakes (DRIs) 2020, Department of Health, Ministry of Public Health as a reference [21]. Due to lack of information on dietary prebiotics and probiotics in the INMUCAL-nutrients software, dietary intakes of prebiotics and probiotics intakes were calculated using nutrition labeling on food product instead.

### Fecal samples

A stool sampling kit consisting of a sample collection tube, sterile wooden tongue depressor and sterile baby pad was given to each participant at 34-36 weeks of gestation. Researcher provide a leaflet providing instruction for fecal collection to participants. According to Bahl et al., fresh feces samples were collected in sterile tubes, transported on ice, and immediately stored at − 20 °C in the laboratory for 7 days before DNA extraction [22].

### DNA extraction

Before the extraction, 0.2 g of stool samples were mechanically homogenized with a Mini BeadBeater 8 (BioSpec, USA) for 4 min at 5,000 rpm according to Smith et al. [23]. After that, genomic DNA from each fecal sample was extracted using a QIAamp DNA stool Mini kit (Qiagen, Hilden, Germany) according to the manufacturer’s instruction. The V3-V4 region of 16S rRNA gene was amplified by polymerase chain reaction (PCR) using the extracted DNA as template and universal primer (16S Amplicon PCR Forward Primer, 5′-TCGTCGGCAGCGTCAGATGTGTATAAGAGACAGCCTACGGGNGGCWGCAG-3′ and 16S Amplicon PCR Reverse Primer: 5′-GTCTCGTGGGCTCGGAGATGTGTA TAAGAGACAGGACTACHVGGGTATCTAATCC-3′) with barcode sequence. Sequences were generated using the Illumina MiSeq (2 × 300 bp) instrument.

### 16S rRNA gene sequence analysis

The raw sequence data was demultiplexed using the q2-demux plugin, and reads with expected errors (maxEE) higher than 3.0 was discarded by denoising software, DADA2 pipeline (version 1.10) (via q2-dada2) [24]. A phylogeny was constructed using the SEPP q2-plugin, placing short sequences into sepp-refs-gg-13-8.qza reference phylogenetic tree [25]. Alpha diversity (observed_OTUs, Faith_PD, and Shannon) and beta diversity based on UNIFRAC distance (phylogenetic) and Bray Curtis distance (non-phylogenetic) are obtained. High-quality sequences were estimated using q2-diversity after samples were rarefied (subsampled without replacement) to 11,478 reads per sample. Taxonomy was identified using the QIIME 2 classifier (version 2020.8) with the Greengenes version 13.8 database [26]. Permutation multivariate analysis of variance-PERMANOVA with 999 permutation and Kruskal-Wallis were used to test the significance of alpha and beta diversity, respectively. Principal component analysis (PCA) and distance-based redundancy analysis (dbRDA) with 999 permutations were performed using the RDA function in the Vegan package of R version 3.5.3.

### Linear discriminant analysis effect size (LEfSe)

LEfSe algorithm (found in the online interface Galaxy: https:// huttenhower. sph.harvard.edu) was used for identification of significant differences in relative abundance of bacterial taxonomy. A LEfSe analysis was performed using phylum to genus-level data in order to identify the relevant taxa responsible for the differences found in pregnant women in the study regions. For the LEfSe analysis, the α value for the factorial Kruskal-Wallis test in the one-against-all strategy was set at 0.05. OTUs with an average abundance was greater than 0.1% in all samples. The abundances were normalized to the sum of 1 million values in each sample. Then linear discriminant analysis (LDA) is performed. The threshold of the logarithmic LDA score was set at 2.0 for discriminative features.

### Statistical analysis

We compared demographic data and dietary habits of pregnant women by regions using Chi-square test for categorical variable and ANOVA test or Kruskal-Wallis test for continuous variables. Differences in relative abundance of each taxa among the subjects in the three regions were analyzed using LEfSe analysis as above mentioned. Differences in the community structures between samples were calculated with weighted Unifrac distance. Based on the Unifrac distance matrix, PERMANOVA analysis was performed with 999 permutations to evaluate significance of differences among the microbiome community.

R is a software environment for statistical analyses and graphics. To examine the association of main dietary intake and the beta diversity of microbiome, the envfit function was performed on the PCA ordination with 999 permutation. The beta diversity explained by the foods and/or residential regions was estimated by the dbRDA function in the R vegan package and R^2^ and *p*-value were estimated by the ‘RsquareAdj’ and ‘Anova’ functions in the R vegan package version 3.5.3. Correlation between glutinous rice intake and abundance of each bacteria taxon was analyzed by Spearman’s rank test.

## Results

There were 86 pregnant women enrolled in this study (11, 65, and 10 from Northern, Central, and Northeastern regions, respectively). The demographic data are shown in Table 1. There was no significant difference in these data among different regions.

**Table 1:** Baseline characteristics of pregnant women categorized by regions

Characteristics	Regions^a^ (*n* = 86)				*p*-value^c,d^
	Total	Northern	Central	Northeastern	
No. of participants	86	11	65	10	
Maternal age, yr	30.0 ± 5.8	29.6 ± 3.9	30.3 ± 6.3	30.0 ± 5.8	0.642
Maternal height, cm	160.6 ± 5.3	160.5 ± 4.0	161.0 ± 5.6	160.6 ± 5.3	0.165
Pre-pregnancy weight, kg	54.4 ± 8.3	52.3 ± 6.5	54.9 ± 8.5	54.4 ± 8.3	0.597
Pre-pregnancy BMI, kg/m^2^	21.1 ± 3.2	20.3 ± 2.6	21.2 ± 3.3	21.1 ± 3.2	0.661
Gestational weight gain, kg	13.9 ± 4.1	14.4 ± 3.4	13.8 ± 4.2	13.9 ± 4.1	0.895
Parity^b^	2 (1, 2)	2 (1, 2)	1 (1, 2)	2 (1, 2)	0.088
Maternal education, n (%)					0.651
Elementary school	10 (11.6)	3 (27.2)	5 (7.7)	2 (20.0)	
High school	14 (16.3)	2 (18.2)	10 (15.4)	2 (20.0)	
Diploma	30 (34.9)	5 (45.5)	22 (33.9)	3 (30.0)	
Bachelor	32 (37.2)	1 (9.1)	28 (43.1)	3 (30.0)	
Household income per mo, n (%)					0.705
≤10,000 baht	16 (18.6)	3 (27.3)	12 (18.5)	1 (10.0)	
10,001 - 20,000 baht	34 (39.5)	4 (36.4)	26 (40.0)	4 (40.0)	
20,001 - 30,000 baht	29 (33.7)	3 (27.3)	22 (33.9)	4 (40.0)	
> 30,000 baht	7 (8.1)	1 (9.1)	5 (7.7)	1 (10.0)	
Maternal antibiotic use during last month of pregnancy, n (%)	5 (6.5)	1 (9.1)	3 (4.6)	1 (10.0)	0.314
Prolonged rupture of membranes, n (%)	0 (0)	0 (0)	0 (0)	0 (0)	1.00
History of allergy, n (%)	8 (10.4)	1 (9.1)	6 (9.2)	1 (10.0)	0.251

^a^Data presented as mean ± SD

^b^median (IQR, interquartile ranges)

^c^Chi-square test for categorical data, ^d^ANOVA with Tukey’s post hoc test or Kruskal-Wallis test with Mann-Whitney U test for continuous data

### Overall phylogenetic profiles of gut microbiome in healthy pregnant women

A total of 13,997,843 sequencing reads were generated and clustered into 1819 OTUs. Of total reads, almost all of the OTUs were classified into the level of specific families. Mean relative abundance (%) at the phylum level was as follows: Bacteroidetes (57.8-78.4%), Firmicutes (13.8-32.6%), Proteobacteria (6.4-8.1%), Fusobacteria (0.4-7.6%), Actinobacteria (0.1-1.5%), and others (0.1-0.8%). At the family level, the gut microbiome was generally dominated by Bacteroidaceae (30.5-50.6%), Prevotellaceae (14.9-17.8%), Lachnospiraceae (4.5-12.0%), Ruminococcaceae (2.2-7.3%), and Sutterellaceae (2.9-4.1%). The abundance of gut microbiome genera or phylum was not significantly correlated with either pre-pregnancy BMI or gestational weight gain. Furthermore, alpha diversity was not correlated to pre-pregnancy BMI (observed OTUs: r_s_ = − 0.187, *p* = 0.085; Faith_PD: r_s_ = − 0.173, *p* = 0.111; Shannon: r_s_ = − 0.116, *p* = 0.288) or gestational weight gain (observed OTUs: r_s_ = − 0.054, *p* = 0.619; Faith_PD: r_s_ = − 0.046, *p* = 0.673; Shannon: r_s_ = − 0.031, *p* = 0.774).

### Difference in fecal gut microbiome communities among pregnant women in the northern, central, and northeastern regions

**Fig. 1:**
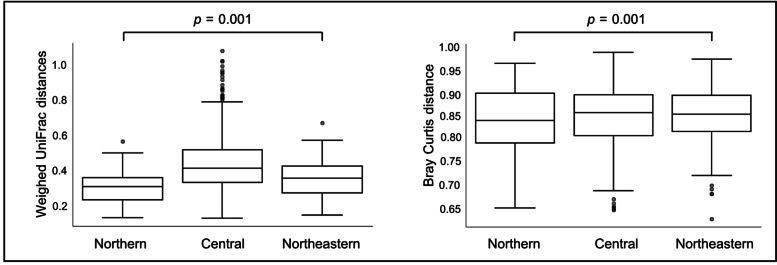
Beta diversities of gut microbiome. The differences between regions were analyzed using the PERMANOVA with 999 permutations and *p*-value is shown over the box plots

**Fig. 2:**
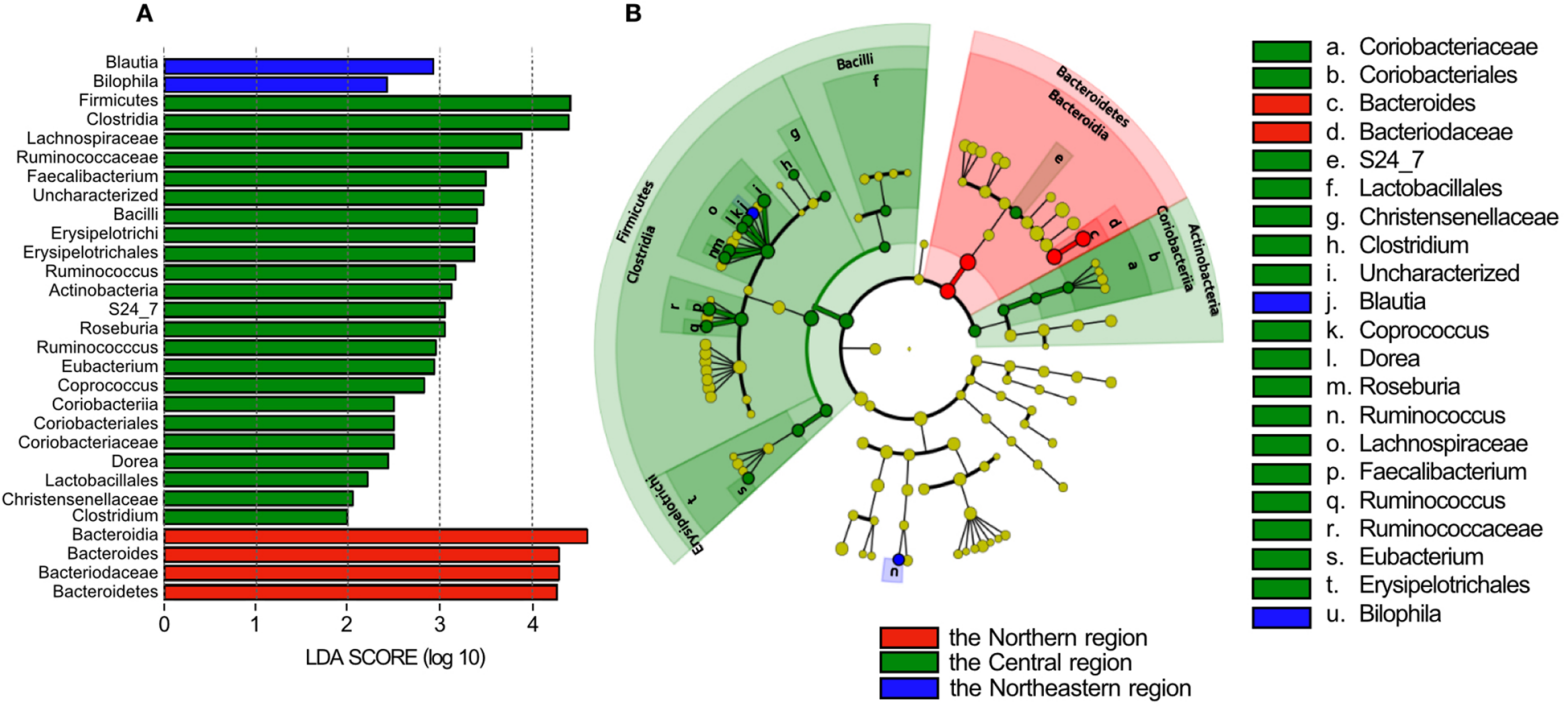
Linear discriminant analysis effect size (LEfSe) analysis identifying differences in abundant taxa in feces of pregnant women in the Northern, Central and Northeastern regions. **A** Taxonomic groups showing LDA scores > 2.0 with *p* < 0.05. **B** Cladogram showing different abundant taxa among feces of pregnant women in the Northern, Central and Northeastern regions (LDA score > 2.0, *p* < 0.05)

We used pairwise weighted UniFrac distances and Bray Curtis distance in Fig. 1 for detect the difference the gut microbiome between region. We found that there were statistical differences in the community structures between Northern, Central and Northeast regions (*p* = 0.001). In addition, a LEfSe analysis showed the statistical differences in the abundance of certain taxonomic groups among the three regions as shown in Fig. 2. The gut microbiome of pregnant women in the Northern region was more highly colonized by phylum Bacteroidetes to genus Bacteroides than that in other regions; whereas the gut of pregnant women in the Central region was more highly colonized by phylum Firmicutes, orders Clostridiales, such as families Ruminococcaceae and Lachnospiraceae, than other regions. Genus *Blautia* and *Bilophila* were enriched in the gut of pregnant women in the Northeastern region.

**Fig. 3:**
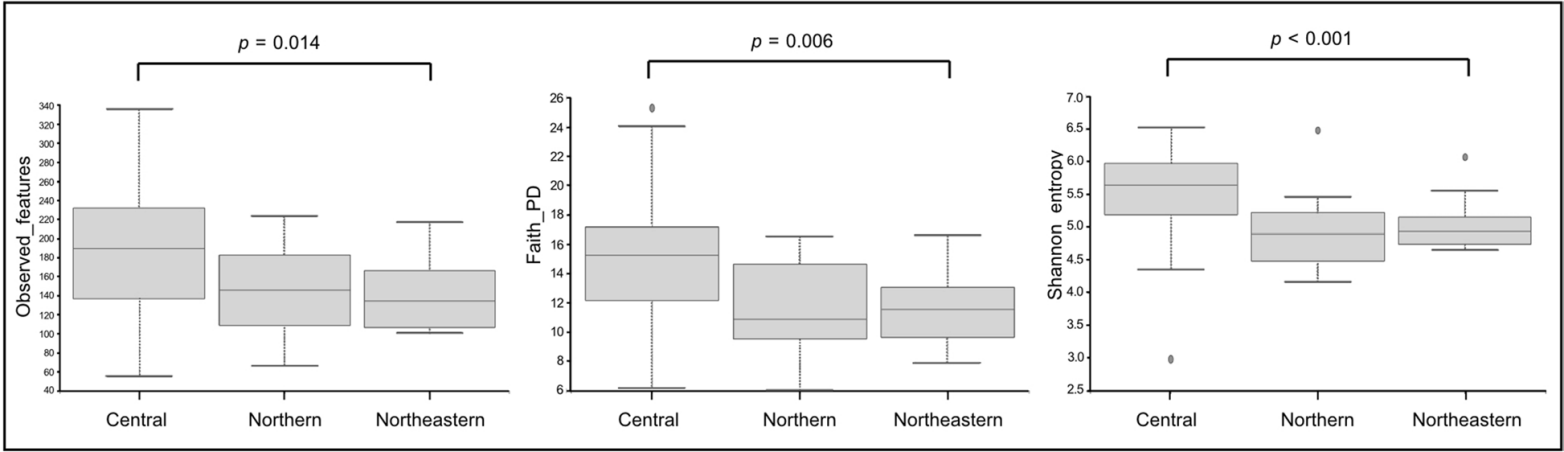
Alpha diversities of gut microbiome. The differences between regions were analyzed using the Kruskal-Wallis test and *p*-value is shown over the box plots

Total alpha diversity tended to be higher in pregnant women in the Central region than that in women in the Northern and Northeastern regions, as indicated by the number of observed OTUs (*p* = 0.0014), Faith_PD (*p* = 0.006), and Shannon (*p* < 0.001). Distribution of these alpha diversity indices in each group of regions are shown in Fig. 3.

### Differences in dietary habits among pregnant women in the northern, central and northeastern regions of Thailand

The median of energy intake in pregnant women was 1955 kcal/day (range 1450-2510), equivalent to 87.44% of Thai DRIs for energy. The energy distribution (%) from carbohydrate: protein: fat was 52: 16: 32, respectively. Which was similar to recommended energy distribution from Thai DRIs [27]. The median intake of protein in participants was 80 g/day (range 72-102), equivalent to 95.24% of Thai DRI for protein. There was no significant difference in the daily energy and nutrient intakes of pregnant women in different regions (Table 2).

**Table 2:** Daily energy and macronutrient intakes of 86 pregnant women according to regions

Nutrient	Region^a^			*p*-value^b^
	Northern (*n* = 11)	Central (*n* = 65)	Northeastern (*n* = 10)	
Energy (kcal)	2015 (1870, 2045)	1946 (1847, 2017)	2021 (1933, 2015)	0.426
Carbohydrate (g)	256 (249, 260)	238 (248, 256)	298 (249, 299)	0.266
Protein (g)	90 (78, 82)	78 (77, 82)	70 (70, 82)	0.627
Fat (g)	69 (58, 75)	75 (58, 78)	61 (60, 77)	0.642
% Energy distribution (CHO: P: F)^c^	51: 18: 31	49: 16: 35	59: 14: 27	

^a^Data presented as median (IQR, interquartile ranges)

^b^The differences between regions were analyzed using the Kruskal-Wallis test

^c^CHO, carbohydrate; P, protein; F, fat

Rice is the main carbohydrate source for Thai people. Based on semi-FFQ data, pregnant women in the Northern region significantly consumed more glutinous rice than those in the Central region but close to those in the Northeastern region (the median were 4, 1, and 3 portions/day, respectively; *p* < 0.001). On the other hand, pregnant women in the Central region significantly consumed more white rice than those in the Northern and the Northeastern regions (the median were 5, 2, and 4 portions/day, respectively; *p* < 0.001).

In addition, vegetables consumption of pregnant women in the Northern region was significantly higher than that of pregnant women in the Central region by about 3 times (*p* = 0.003) but not significantly different from that of those in the Northeastern region (*p* = 0.075).

There was no significant difference in other food items such as bread and cereal, milk product, fruit, fish, sea food, meat, and oil consumption (Table 3).

**Table 3:** Food items from semi-FFQ data of 86 pregnant women according to regions

Food items	Average of food intake (portion/day)^a^			
	Northern (*n* = 11)	Central (*n* = 65)	Northeastern (*n* = 10)	*p*-value^b^
White rice	2 (2, 5)	5 (4, 5)	4 (2, 5)	0.000^**^
Glutinous rice	4 (1, 6)	1 (0, 1)	3 (1, 5)	0.000^**^
Bread & Cereal	1 (1, 2)	2 (1, 2)	1 (0, 2)	0.489
Red meat	2 (2, 3)	4 (2, 4)	2 (2, 3)	0.883
Sea food	1 (1, 2)	1 (0, 2)	1 (0, 2)	0.655
Fish	1 (0, 2)	1 (1, 1)	1 (0, 1)	0.791
Egg	1 (1, 2)	1 (1, 2)	2 (1, 2)	0.194
Milk & Dairy product	1 (1, 2)	3 (1,3)	2 (0, 2)	0.788
Vegetable	3 (2, 4)	1 (1, 2)	2 (2, 3)	0.003^*^
Fruit	1 (0, 2)	1 (1, 2)	1 (0, 1)	0.693
Oil	2 (2, 3)	2 (2, 3)	2 (2, 3)	0.643

^a^Data presented as median (IQR, interquartile range)

^b^The differences between regions were analyzed using the Kruskal-Wallis test

Significant difference is indicated with different asterisk, *p < 0.05, **p < 0.001

### Correlation between dietary nutrients and gut microbiome profile in Thai pregnant women

**Fig. 4:**
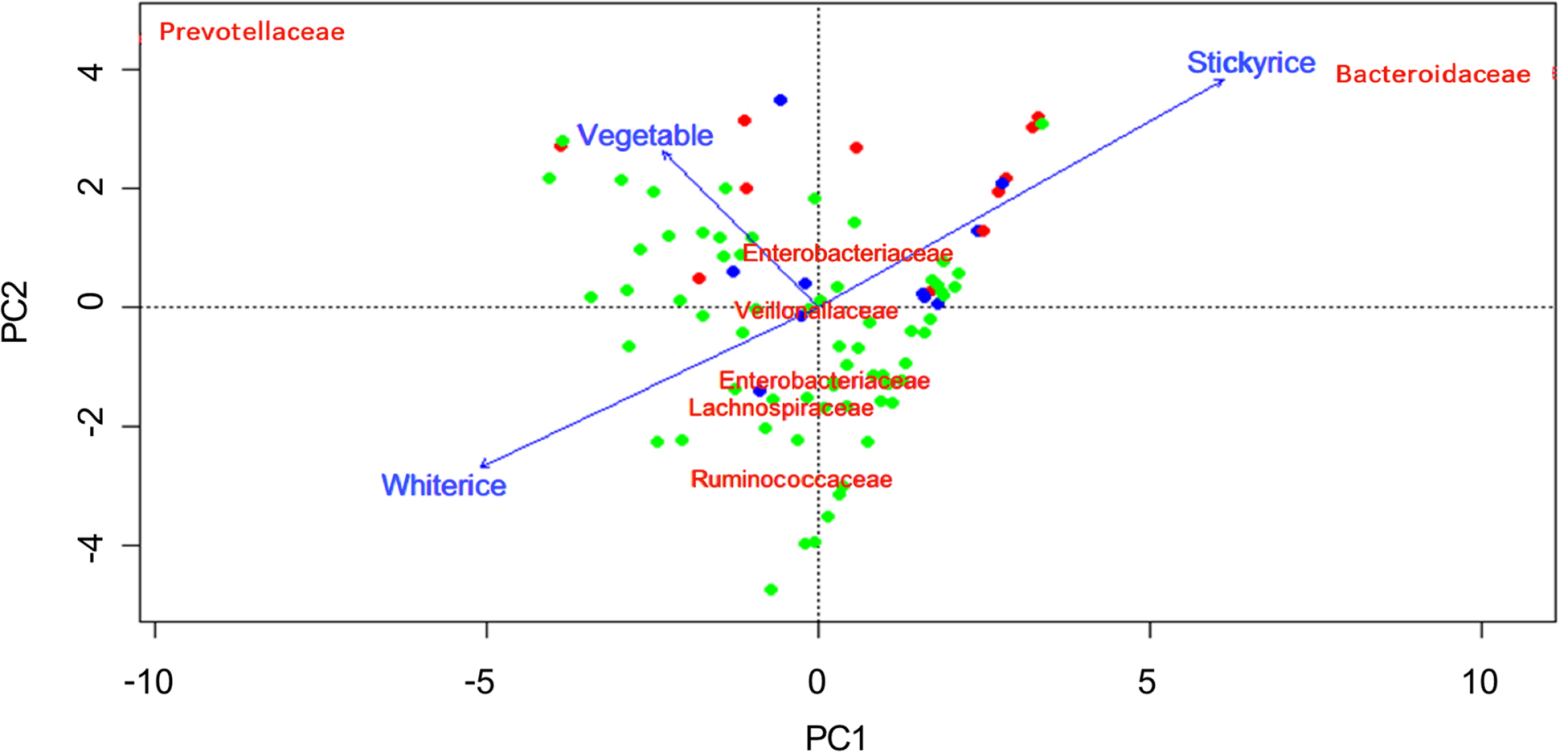
Correlation between dietary intake and gut microbiome communities in pregnant women in 3 regions of Thailand. A principal component analysis (PCA) was performed using microbiome dataset of 86 pregnant women (  , the Northern region;  , the Central region; and  , the Northeastern region) at family level. The consumed amounts of 11 main foods were correlated with the PCA ordination using envfit function with 999 permutation and the foods showing the correlation with *p* < 0.05 were displayed as vectors

RDA analysis indicated that dietary patterns consisting of 11 main food items explained 43.8% of variation of gut microbiome profile in our 86 subjects. In contrast, residential regions explained only 7.0%, suggesting that the consumed foods had dominant impact on gut microbiome. Further, we performed an envfit analysis to extract dominant dietary factors to affect the gut microbiome structure of pregnant women. As shown in Fig. 4, the intake of three food items, namely glutinous rice, white rice, and vegetables, discretely showed a strong significant correlation with gut microbiome variance in pregnant women. Notably, glutinous rice showed very high R^2^ score (0.62) in association with Bacteroidaceae. On the other hand, the correlation vector of white rice oriented opposite to glutinous rice that directed to the family members of Firmicutes group, such as Ruminococcaceae and Lachnospriaceae. Further, vegetable are solely associated with Prevotellaeae.

To determine the correlation between glutinous rice intake and gut microbiome commensals in more detail, Spearman’s correlation coefficient was used for calculation between glutinous rice intake and the relative abundance of each taxonomic group (Table 4). We found that glutinous rice intake was correlated positively with Bacteroidetes and negatively with Firmicutes. The genus *Bacteroides* show strong correlation with glutinous rice intake (rho = 0.738, *p* < 0.001), while the genus *Prevotella* was negatively correlated with glutinous rice intake (rho = − 0.487, *p* < 0.001). For Firmicutes, the families Ruminococcaceae and Lachnospiraceae showed a significantly negative correlation with glutinous rice intake (rho = − 0.315*, p* < 0.05 and − 0.394, *p* < 0.001, respectively). Genus *Enterobacteriaceae*, from the phylum Proteobacteria was not associated with glutinous rice consumption.

**Table 4:** Correlation coefficient between glutinous rice intakes and relative abundance of each taxonomic group

Taxon^a^	rho	%Relative abundance^b^	*p*-value
p_Bacteroidetes	0.405	59.51	0.001
p_Bacteroidetes; c_Bacteroidia	0.405	0.59	0.001
p_Bacteroidetes; c_Bacteroidia; o_Bacteroidales	0.405	0.59	0.001
p_Bacteroidetes; c_Bacteroidia; o_Bacteroidales; f_Bacteroidaceae	0.738	33.70	0.001
p_Bacteroidetes; c_Bacteroidia; o_Bacteroidales; f_Bacteroidaceae; g_Bacteroides	0.738	0.34	0.001
p_Bacteroidetes; c_Bacteroidia; o_Bacteroidales; f_Prevotellaceae	−0.487	16.41	0.001
p_Bacteroidetes; c_Bacteroidia; o_Bacteroidales; f_Prevotellaceae; g_Prevotella	−0.487	0.16	0.001
p_Firmicutes	−0.440	28.72	0.001
p_Firmicutes; c_Clostridia	−0.441	26.60	0.001
p_Firmicutes; c_Clostridia; o_Clostridiales; f_Ruminococcaceae	−0.315	4.90	0.003
p_Firmicutes; c_Clostridia; o_Clostridiales; f_Ruminococcaceae; Other	−0.315	4.90	0.003
p_Firmicutes; c_Clostridia; o_Clostridiales; f_Lachnospiraceae	−0.394	3.00	0.001
p_Firmicutes; c_Clostridia; o_Clostridiales; f_Lachnospiraceae; g_Blautia	−0.201	0.80	0.064

^a^p, phylum; c, class; o, order; f, family; g, genus

^b^Mean relative abundance in 86 pregnant women

## Discussion

This study was the first study to compare gut microbiome profiles of Thai pregnant women from different regions with different dietary cultures. We found that the overall microbiome composition in pregnant women in their third trimester resembled the typical composition in healthy Thai adults [28, 29], with a dominance of taxa from the phylum of Bacteroidetes, Firmicutes, and Proteobacteria. However, Koren et al. reported that gut microbiome profiles significantly changed from the first to the third trimesters. There was an increase in predominance of Proteobacteria and Actinobacteria but a decrease in alpha diversity with advancing gestational age [3]. In a prospective case-control study of 40 pregnant women, DiGiulio et al. analyzed microbiome composition of 3767 specimens collected weekly during pregnancy and monthly after delivery. They found no dramatic change during gestation or upon delivery in gut microbiome composition or richness indexes [5]. However, the results from the present study cannot conclude the possibility of change in the taxonomic structure of gut microbiome profiles when pregnancy progressed for we obtained samples only one time in the third trimester.

The maternal microbiome during pregnancy impact on pregnancy outcomes and infant health, including cardiometabolic complications of pregnancy such as preeclampsia and gestational hypertension, gestational weight gain, and preterm delivery [30]. Pregnant women with gestational diabetes had the least gut microbiome diversity during the first trimester [3]. A few studies have established the role of gut microbiome concerning gestational hypertension and preeclampsia. Amarasekara et al. demonstrated the presence of microorganism in amniotic fluid in pregnant women with preeclampsia [31].

In addition, pre-pregnancy obesity and maternal excess weight gain increase the risk of fetal macrosomia, cesarean delivery, neonatal hyperinsulinemia, and metabolic syndrome in childhood. In overweight or obese pregnant women, a healthy gestational weight gain is associated with a significantly lower risk of preeclampsia, cesarean delivery, and large for gestational age birth [32]. Stanislawski et al. found that pre-pregnancy overweight or obesity is associated with reduced alpha diversity in mothers and differences in microbial composition [33]. In the present study, alpha diversity was not correlated to the pre-pregnancy BMI or gestational weight gain.

Diet has been shown to affect the gut microbiome. A few research studies have examined diet during pregnancy with respect to the gut microbiome. Recently, a systematic review showed the association between the maternal diet and gut microbiome. High-fat diets, fat-soluble vitamins and fiber consumed by pregnant women were the most significant diets associated with gut microbiome composition of both infants and mothers. High-fat diets were significantly associated with reduced microbial diversity. Fiber was positively related to microbial diversity [34].

In the present study, we collected dietary data from 3-day food records and semi-FFQ data. We found no statistically significant difference in daily energy and nutrient intakes of pregnant women from different regions. All pregnant women in three regions consumed rice, particularly steamed rice. When categorized by type of rice, pregnant women in the Northern region consumed glutinous rice in a similar portion to those in the Northeastern region; their consumption of glutinous rice was higher than that in those in the Central region. The results might be attributed to the fact that people of the Northern region prefer glutinous rice to white rice, rolling them into balls with their hands and dipping them in the dipping sauces. In the Central region, Thai staples and side dishes are being replaced by diets with higher proportions of fats and animal meat but fewer vegetables and fruits. Concomitant with these trends is the selection of foods that require less time and skill to prepare. Our results suggested that food consumption by pregnant women from each region is different. This can be an important environmental factor that shapes the gut microbiome in pregnant women.

Glutinous rice (Oryza glutinosa L.), is one of the main sources of carbohydrates in Thailand. It serves as a staple food in the Northern and Northeastern regions of Thailand and in parts of Laos and Cambodia. The glutinous rice absorbs about half as much water as regular rice with a sticky texture when cooked. Glutinous rice is known for its very low amylose and high amylopectin contents [35], giving unique eating quality characteristics to processed products [36].

Amylopectin is a highly branched polymer containing many short-chained branches with a high molecular weight of 10^7 − 9^ Da [37], making up approximately 35% by weight of total grain composition. In 2013, Jiang et al. conducted an in vitro study to compare the fermentation characteristics of amylopectin and resistant starch by the colonic microbiota of pigs. They demonstrated changes in the composition of bacterial communities during the fermentation [38]. Furthermore, their study showed that amylopectin was more fermentable by colonic microbiota, while resistant starch in that study showed very poor fermentation characteristics.

Martínez et al. reported a double-blind, crossover study in 10 human subjects consuming crackers containing either RS2, RS4, or native starch, for 3 weeks. They found that RS4, not RS2 induced changes in the phylum level of the gut microbiome by significantly increasing Actinobacteria and Bacteroidetes but decreasing Firmicutes [39]. This fact supports that amylopectin is probably the principal promotor for *Bacteroides* spp.

In the present study, pregnant women in the Northern region consumed glutinous rice more than those in other regions, and they had a high abundance of Bacteroidetes. Amylopectin in glutinous rice may help promote the growth of Bacteroidetes in the gastrointestinal tract of pregnant women. However, this hypothesis cannot be proven in the present study. The present study has limitations due to the relatively small sample size, no data of microbiome profile of pregnant women in the Southern region, as well as lack of microbial function and biochemical information. Further studies are needed to demonstrate and understand the mechanical property of amylopectin and its contribution to promoting the growth of Bacteroidetes.

## Conclusions

The results from the present study suggest that gut microbiome profiles in pregnant women are associated with distinct main carbohydrate consumption, with Bacteroidetes and Firmicutes being prominent gut microbiome. Glutinous rice is related to abundance of Bacteroidetes. Further study is needed to understand how Bacteroides species dominate microbiome of pregnant women consuming glutinous rice.

## Supplementary Information

**Additional file 1.**

## Data Availability

The datasets generated and/or analyzed during the current study are available from the corresponding author on reasonable request.

## Abbreviations

BMI: Body mass index
FFQ: Food frequency questionnaires
DRIs: Dietary reference intakes
PCR: Polymerase chain reaction
OTUs: Operational taxonomic units
PD: Phylogenetic diversity
PCA: Principal component analysis
dbRDA: Distance-based redundancy analysis
LEfSe: Linear discriminant analysis effect size
LDA: Linear discriminant analysis
