# Supplementary material for: Gut microbiome profiles in Thai healthy pregnant women and its association with types of foods
Source: BMC Pregnancy Childbirth. 2022 Jan 29;22:79. doi: 10.1186/s12884-022-04397-5 (PMC8801080; doi:10.1186/s12884-022-04397-5)
Supplement: Supplementary file 1 — Additional file 1. [file 12884_2022_4397_MOESM1_ESM.docx]

**Food Frequency Questionnaire**

**Instructions:** This questionnaire is about your usual eating habits over the month. Where possible give only one answer per question for the type of food you eat most often or choose rarely/never, when applicable.

**Breads and Cereals**

| **Food item** | | **Amount eaten** | **Number of times eaten** | | | |
| --- | --- | --- | --- | --- | --- | --- |
| **How often do you eat the following:** | |  | **Per day** | **Per week** | **Per month** | **Rarely/Never** |
| **Bread** | | | | | | |
| 1 | White bread |  |  |  |  |  |
| 2 | Wholemeal bread |  |  |  |  |  |
| 3 | Bread with fruits and nuts |  |  |  |  |  |
|  | **Bread spreads used** |  |  |  |  |  |
| 4 | Butter |  |  |  |  |  |
| 5 | Margarine |  |  |  |  |  |
| 6 | Peanut butter |  |  |  |  |  |
| 7 | Jams/honey |  |  |  |  |  |
|  | **Other types of breads** |  |  |  |  |  |
| 8 | Bread buns with coconut/meat fillings |  |  |  |  |  |
|  | **Cereal** |  |  |  |  |  |
| 9 | Plain/flavored breakfast cereal |  |  |  |  |  |
| 10 | Mixed (with fruit/nuts) breakfast cereal |  |  |  |  |  |
| 11 | whole-grains breakfast cereals |  |  |  |  |  |
| 12 | Oats/oatmeal (raw) |  |  |  |  |  |
| 13 | Others (pls specify:________) |  |  |  |  |  |
| **Rice and Porridge** | | | | | | |
| 1 | White rice |  |  |  |  |  |
| 2 | Brown or red rice |  |  |  |  |  |
| 3 | Plain porridge |  |  |  |  |  |
| 4 | Fried rice |  |  |  |  |  |
| 5 | Glutinous rice |  |  |  |  |  |
| 6 | Flavoured porridge (e.g. chicken, pork, duck, fish) |  |  |  |  |  |
| 7 | Others (pls specify:________) |  |  |  |  |  |
| **Other sources of carbohydrate** | | | | | | |
| 1 | Corn |  |  |  |  |  |
| 2 | Cassava |  |  |  |  |  |
| 3 | Sweet potatoes |  |  |  |  |  |
| 4 | Others (pls specify : _______ ) |  |  |  |  |  |
| **Fermented food** | | | | | | |
| 1 | Fermented fish |  |  |  |  |  |
| 2 | Ka-pi |  |  |  |  |  |
| 3 | Tempe |  |  |  |  |  |
| 4 | Kimchi |  |  |  |  |  |
| 5 | Nato |  |  |  |  |  |
| 6 | Kaomark (sweet fermented rice) |  |  |  |  |  |
| 7 | Others (pls specify: _______________________ ) |  |  |  |  |  |
| **Noodles (rice noodles, wheat noodles, bean noodles, pasta)** | | | | | | |
| 1 | Rice noodle (size....................) |  |  |  |  |  |
| 2 | Wheat- based soup noodles (e.g. mee, udon, ramen, mee rebus) |  |  |  |  |  |
| 3 | Fried rice noodle |  |  |  |  |  |
| 4 | Fried wheat noodle |  |  |  |  |  |
| 5 | Fermented rice noodles (Kanom-jeen) |  |  |  |  |  |
| 6 | Instant/cup noodles |  |  |  |  |  |
| 7 | Boiled noodles/spaghetti/pasta (plain) |  |  |  |  |  |
| 8 | Boiled noodles/spaghetti/pasta with creamy white sauce |  |  |  |  |  |
| 9 | Others  (pls specify: _____________) |  |  |  |  |  |
| **Soups** | | | | | | |
| 1 | Cream soup |  |  |  |  |  |
| 2 | Clear soup/broth |  |  |  |  |  |
| **Vegetables and Bean curd** | | | | | | |
| 1 | Stir fried, plain |  |  |  |  |  |
| 2 | Stir fired, with meat/seafood |  |  |  |  |  |
| 3 | Stir fried in oyster source |  |  |  |  |  |
| 4 | Curry/Lemak |  |  |  |  |  |
| 5 | Raw/steam/in soup |  |  |  |  |  |
| 6 | Stir fried, plain |  |  |  |  |  |
| 7 | Stir fried, with meat/seafood |  |  |  |  |  |
| 8 | Stir fried in oyster sauce |  |  |  |  |  |
| 9 | Dried legumes (e.g. dhal, dried beans) in gravy |  |  |  |  |  |
| 10 | Raw/steamed/boiled |  |  |  |  |  |
|  | **Mixed vegetables** |  |  |  |  |  |
| 11 | Stir fried, plain |  |  |  |  |  |
| 12 | Stir fried, with meat/seafood |  |  |  |  |  |
| 13 | Stir fried in oyster sauce |  |  |  |  |  |
| 14 | Battered deep fried (e.g. tempura) |  |  |  |  |  |
| 15 | Curry/Lemak |  |  |  |  |  |
| 16 | Raw/steamed/in soup/rojak |  |  |  |  |  |
| 17 | Fried |  |  |  |  |  |
| 18 | Steamed/in soup |  |  |  |  |  |
| 19 | Stir fried potatoes |  |  |  |  |  |
| 20 | Soups with meat stock |  |  |  |  |  |
| 21 | Stews |  |  |  |  |  |
| 22 | Preserved vegetable |  |  |  |  |  |
| **Salad dressings** | | | | | | |
| 1 | Creamy dressing-regular (e.g thousand island, mayonnaise, salad cream etc) |  |  |  |  |  |
| 2 | Creamy dressing-light/low fat |  |  |  |  |  |
| 3 | Oil-based dressing |  |  |  |  |  |
| 4 | Others  (pls specify :_____________ ) |  |  |  |  |  |
| **Fruits** | | | | | | |
| 1 | Orange/red/yellow fresh fruits |  |  |  |  |  |
| 2 | Other fresh fruits |  |  |  |  |  |
| 3 | Fresh fruit juice |  |  |  |  |  |
| 4 | Bananas |  |  |  |  |  |
| 5 | Canned fruits |  |  |  |  |  |
| 6 | Mixed fruits (dried) |  |  |  |  |  |
| 7 | Others  (pls specify: _____________ ) |  |  |  |  |  |
| **Poultry** | | | | | | |
| 1 | Stir fried |  |  |  |  |  |
| 2 | Pan/deep fried |  |  |  |  |  |
| 3 | Coconut curry |  |  |  |  |  |
| 4 | Curry without coconut |  |  |  |  |  |
| 5 | Stew/braised/roasted |  |  |  |  |  |
| 6 | Steamed |  |  |  |  |  |
|  | **Poultry-with skin** |  |  |  |  |  |
| 7 | Stir fried |  |  |  |  |  |
| 8 | Pan/deep fried |  |  |  |  |  |
| 9 | Coconut curry |  |  |  |  |  |
| 10 | Curry without coconut |  |  |  |  |  |
| 11 | Stew/braised/roasted |  |  |  |  |  |
| 12 | Steamed |  |  |  |  |  |
| **Meat** | | | | | | |
|  | **Meat- lean** |  |  |  |  |  |
| 1 | Stir fried |  |  |  |  |  |
| 2 | Pan/deep fried |  |  |  |  |  |
| 3 | Coconut curry/rending |  |  |  |  |  |
| 4 | Curry without coconut |  |  |  |  |  |
| 5 | Stewed/braised |  |  |  |  |  |
| 6 | Roasted/grilled/BBQ |  |  |  |  |  |
| 7 | Steamed/soup |  |  |  |  |  |
|  | **Meat- lean and fat** |  |  |  |  |  |
| 8 | Stir fried |  |  |  |  |  |
| 9 | Pan/deep fried |  |  |  |  |  |
| 10 | Coconut curry/rending |  |  |  |  |  |
| 11 | Curry without coconut |  |  |  |  |  |
| 12 | Stewed/braised |  |  |  |  |  |
| 13 | Roasted/grilled/BBQ |  |  |  |  |  |
| 14 | Steamed/soup |  |  |  |  |  |
| **Fish/Seafood** | | | | | | |
| 1 | Raw (e.g. sashimi) |  |  |  |  |  |
| 2 | Stir fried/pan fried/deep fried |  |  |  |  |  |
| 3 | Steamed |  |  |  |  |  |
| 4 | With coconut milk/meat |  |  |  |  |  |
| 5 | Grilled |  |  |  |  |  |
| 6 | Canned (e.g. tuna) |  |  |  |  |  |
| **Other seafood** | | | | | | |
| 1 | Raw (e.g. sashimi) |  |  |  |  |  |
| 2 | Stir fried/pan fried/deep fried |  |  |  |  |  |
| 3 | Steamed |  |  |  |  |  |
| 4 | With coconut milk/meat |  |  |  |  |  |
| 5 | Grilled |  |  |  |  |  |
| 6 | Canned (e.g. tuna) |  |  |  |  |  |
| **Processed foods** | | | | | | |
| 1 | Sausages |  |  |  |  |  |
| 2 | Bacon |  |  |  |  |  |
| 3 | Ham |  |  |  |  |  |
| 4 | Fish cakes |  |  |  |  |  |
| 5 | Fish balls |  |  |  |  |  |
| 6 | Canned meat |  |  |  |  |  |
| 7 | Others  (pls specify: _____________) |  |  |  |  |  |
| **Eggs** | | | | | | |
|  | **Whole eggs (including salted and century eggs)** |  |  |  |  |  |
| 1 | Boiled/poached/in soup/steamed |  |  |  |  |  |
| 2 | Fried/scrambled |  |  |  |  |  |
| **Desserts/Local Snacks** | | | | | | |
|  | **Dessert with coconut milk** |  |  |  |  |  |
| 1 | Banana in coconut milk |  |  |  |  |  |
| 2 | Sweet pumpkin in coconut milk |  |  |  |  |  |
| 3 | Others  (pls specify: _____________) |  |  |  |  |  |
|  | **Glutinous rice in coconut milk** |  |  |  |  |  |
| 4 | Glutinous rice steamed with banana and coconut milk |  |  |  |  |  |
| 5 | Glutinous rice with custard |  |  |  |  |  |
| 6 | Others  (pls specify : _____________) |  |  |  |  |  |
|  | **Others** |  |  |  |  |  |
| 7 | Fried snacks (e.g. Pa-tong-ko, banana chips) |  |  |  |  |  |
| 8 | Dim Sum- steamed (e.g. chee cheong fun, dumplings, rice dumplings) |  |  |  |  |  |
| 9 | Others  (pls specify:______________) |  |  |  |  |  |
| **Biscuits, Pastries and Cakes** | | | | | | |
| 1 | Plain biscuits |  |  |  |  |  |
| 2 | Cream filled biscuits/shortbread |  |  |  |  |  |
| 3 | Puff/flaky pastries (croissants, baked curry puffs etc) |  |  |  |  |  |
| 4 | Plain butter cake/fruit cake |  |  |  |  |  |
| 5 | Sponge cakes |  |  |  |  |  |
| 6 | Cream cakes |  |  |  |  |  |
| **Fast Foods** | | | | | | |
| 1 | Burgers, with beef or chicken |  |  |  |  |  |
| 2 | Burgers, fish |  |  |  |  |  |
| 3 | French fries |  |  |  |  |  |
| 4 | Pizza |  |  |  |  |  |
| 5 | Mashed potato with gravy |  |  |  |  |  |
| **Sweetened beverages** | | | | | | |
| 1 | Sweetened beverages (e.g. soft drinks, packet drinks, yoghurt drinks) |  |  |  |  |  |
| **Nuts** | | | | | | |
|  | **All types of nuts** |  |  |  |  |  |
| 1 | Dry roasted |  |  |  |  |  |
| 2 | Fried |  |  |  |  |  |
| **Snacks** | | | | | | |
| 1 | Fried salty snacks (crisps, prawn crackers, keropok, salted biscuits etc) |  |  |  |  |  |
| 2 | Ice cream |  |  |  |  |  |
| 3 | Chocolate |  |  |  |  |  |
| 4 | Others  (pls specify: _____________) |  |  |  |  |  |
| **Beverages** | | | | | | |
| 1 | Coffee without sugar |  |  |  |  |  |
| 2 | Coffee with sugar |  |  |  |  |  |
| 3 | Tea without sugar |  |  |  |  |  |
| 4 | Tea with sugar |  |  |  |  |  |
| 5 | Malt beverages (e.g. hot chocolate, Milo, Ovaltine) |  |  |  |  |  |
| 6 | Others  (pls specify: _____________) |  |  |  |  |  |
| **Milk & Dairy Products** | | | | | | |
|  | **Milk (as a drink)** |  |  |  |  |  |
| 1 | Full cream milk (fresh, UHT, powder) |  |  |  |  |  |
| 2 | Low fat milk (fresh, UHT, Powder) |  |  |  |  |  |
| 3 | Skimmed milk (fresh, UHT, powder) |  |  |  |  |  |
| 4 | **Yoghurt** |  |  |  |  |  |
| 5 | Regular |  |  |  |  |  |
| 6 | Low fat (including frozen yoghurt) |  |  |  |  |  |
| 7 | Kerds, Kefir |  |  |  |  |  |
| **Cheese** | | | | | | |
| 1 | Cheese/cheese spread |  |  |  |  |  |
| 2 | Low fat cheese |  |  |  |  |  |
| 3 | Others  (pls specify: _____________) |  |  |  |  |  |
| **Soya Products** | | | | | | |
| 1 | Soya milk (fresh/packet/can) |  |  |  |  |  |
| 2 | Soya beancurd (tau huay) |  |  |  |  |  |
| 3 | Tofu |  |  |  |  |  |
| 4 | Others  (pls specify: _____________) |  |  |  |  |  |
| **Vegetarian (Chinese)** | | | | | | |
| 1 | Fried vegetarian noodles |  |  |  |  |  |
| 2 | Gluten (mock char siew/duck) |  |  |  |  |  |
| 3 | Fried bean curd sheet |  |  |  |  |  |
| 4 | Others  (pls specify: _____________) |  |  |  |  |  |

Other frequently consumed foods (which do not state in the above-mentioned list but you consume quite frequently).

| **Food item** | | **Amount eaten** | **Number of times eaten** | | | |
| --- | --- | --- | --- | --- | --- | --- |
| **How often do you eat the following:** | |  | **Per day** | **Per week** | **Per month** | **Rarely/Never** |
|  |  |  |  |  |  |  |
|  |  |  |  |  |  |  |
|  |  |  |  |  |  |  |
|  |  |  |  |  |  |  |
|  |  |  |  |  |  |  |
